# Plagiarism and unethical practices in literature

**Published:** 2009

**Authors:** Rajesh Sinha, Gurnarinder Singh, Chandrashekhar Kumar

## Introduction

Plagiarism is defined as “the unauthorized use or close imitation of the language and thoughts of another author and the representation of them as one's own original work”. In the academic world, plagiarism is a very serious offense that can result in punishments such as a suspension or expulsion. Plagiarism can vary in its extent based on the amount of plagiarism. Minimal amount of plagiarism is very common in the educational sector where person do plagiarism by substituting the synonyms and editing the original text. Sometimes complete plagiarism is seen where one presents the work without making any change in the data and presents it as one's own work. Apart from plagiarism, falsification and fabrication of data also constitute serious offense. Falsification and fabrication call into question the integrity of data and the data record. Practice of omitting or altering research materials, equipment, data, or processes in such a way that the results of the research are no longer accurately reflected in the research result is called falsification whereas the practice of inventing data or results and recording them in the research record is called fabrication. Both of these affect the credential of the research. Double publication” is a practice which involves repeat publication, or attempts at publication, of text, figures, or data in any form of publicly available media without citation in the later manuscript. All these things including plagiarism, falsification and fabrication, double publications are serious transgression of academic ethics.

In the present article, we have made an attempt to review the literature related to such acts.

### Plagiarism

**McCabe *et al.*** (Mem Cognate 2007;35(2):231-41) reported two experiments examining inadvertent plagiarism in young and older adults. Young and older adults took turns generating category exemplars in small groups, and after a short retention interval recall was tested and subjects were asked to generate new exemplars (i.e., exemplars not initially generated). When asked to generate new exemplars, older adults were more likely to repeat exemplars that had been generated earlier by others (i.e., generate-new plagiarism). When asked to recall the exemplars they had generated earlier, older adults were more likely to claim that they had generated exemplars that had been generated by others (i.e., recall-own plagiarism), and were also more likely to falsely recall exemplars that had not been generated at all. There were no age differences in confidence for items that were plagiarized on the generate-new task. Hierarchical regression analyses indicated that age differences in generate-new plagiarism and false recall were entirely mediated by measures of episodic recall and working memory capacity. They concluded that inadvertent plagiarism errors result from the failure of systematic decision processes, and that controlled attention is important for avoiding memory errors.

**Cole *et al.*** (Fam Med. 2007;39(6):436-8) opined that the act of overt plagiarism by graduates of accredited residency programs represents a failure in personal integrity. It also indicates a lack of professionalism. A recent experience at one geriatric fellowship indicated that the problem of plagiarism might be more prevalent than previously recognized. A situation was discovered at the geriatric medicine fellowship at Florida Hospital Family Medicine Residency Program in Orlando, in which three of the personal statements included in a total of 26 applications to the fellowship in the past 2 years contained portions plagiarized from a single web site. The aim in documenting this plagiarism was to raise awareness among medical educators about the availability of online sources of content and ease of electronic plagiarism. Some students and residents may not recognize copying other resources verbatim as plagiarism. The authors felt that residency programs should evaluate their own need for education about plagiarism and include this in the training of the competency of professionalism.

**Harper** (Nurse Educ Today 2006;26(8):672-9) noted that the use of technology has enhanced the convenience, flexibility, and efficiency of both preparatory and continuing education. Unfortunately, academic dishonesty, including plagiarism, has shown a positive correlation with the increased use of technology in education. A review of the literature related to unintended outcomes of the use of technology in nursing education and continuing education was conducted to determine the ethical implications for the nursing profession. Although nursing research dealing with academic and professional misconduct is sparse, evidence suggests that academic dishonesty is a predictor of workplace dishonesty. Given this correlation between unethical classroom behavior and unethical clinical behavior, efforts to staunch academic dishonesty may help allay professional misconduct. They concluded that a combination of high tech and low tech methods may be used to minimize unethical behaviors among students and practicing professional nurses in order to maintain the integrity of the profession.

**Bassendowski** (Int J Nurs Educ Scholarsh. 2005; 2:Article 3. Epub 2005 Feb 25) observed that with the reports of plagiarism in post-secondary institutions and the ease with which students can ‘cut and paste’ content from online sources, the relevance and applicability of traditional assessment strategies need to be examined in light of these technological advances. The paper explores a connection to the visual arts in terms of creation, re-creation, the ‘desire to conceal’, and contemporary means of interpretation.

**Logue** (Nurs Stand. 2004;18(51):40-3) examined the issue of plagiarism by nursing students and academics in British universities and highlighted how electronic developments such as the internet and word processing have made it easier. It describes how some websites support plagiarism and how, for a price, a qualification up to and including higher degree level may be gained without the recipient of the award having to do any coursework.

**Perfect *et al.*** (Memory 2008;16(4):386-94) observed that when groups of individuals work together to generate solutions to a problem, one member of the group can plagiarise another either by recalling that person's idea as their own (recall-own plagiarism), or by generating a novel solution that duplicates a previous idea (generate-new plagiarism). This study examined the extent to which these forms of plagiarism are influenced by the quality of the ideas. Groups of participants initially generated ideas, prior to an elaboration phase in which idea quality was manipulated in two ways: participants received feedback on the quality of the ideas as rated by independent judges, and they generated improvements to a subset of the ideas. Unconscious plagiarism was measured in recall-own and generate-new tasks. For recall, idea improvement led to increased plagiarism, while for the generate-new task, the independent ratings influenced plagiarism. These data indicated that different source-judgement processes underlie the two forms of plagiarism, neither of which can be reduced simply to memory strength.

### Double Publication

**Kostoff** (Sci Eng Ethics 2006;12(3):543-54) examined the similarity of documents in a large database of published Fractals articles for redundancy. Three different text matching techniques were used on published abstracts to identify redundancy candidates, and predictions were verified by reading full text versions of the redundancy candidate articles. A small fraction of the total articles in the database was judged to be redundant. This was viewed as a lower limit, because it excluded cases where the concepts remained the same, but the text was altered substantially. Far more pervasive than redundant publications were publications that did not violate the letter of redundancy but rather violated the spirit of redundancy. There appeared to be widespread publication maximization strategies. Studies that resulted in one comprehensive paper decades ago now result in multiple papers that focus on one major problem, but are differentiated by parameter ranges, or other stratifying variables. This ‘paper inflation’ is due in large part to the increasing use of metrics (publications, patents, citations, etc) to evaluate research performance, and the researchers' motivation to maximize the metrics.

**Roig** (Psychol Rep. 2005;97(1):43-9) performed a preliminary, two-part study exploring the extent to which authors reuse portions of their own text from previously published papers. All 9 articles from a recent issue of a psychology journal were selected as target papers. Up to 3 of the most recent references cited in each of the target articles and written by the same authors were also obtained. All target articles and their corresponding references were stored digitally. Then, using specialized software, each reference was compared to its target article to assess the number of strings of text identical to both papers. Only one of the nine target articles reused significant amounts of text from one of its references. To explore further the possibility of additional text reuse, the references in each of the 9 sets of papers were compared against each other. The new comparison identified 5 pairs of papers with a substantial number of identical strings of text of 6 consecutive words in length or longer, but most of the reused text was confined to the Method section. The results suggested that some of these authors reused their own text with some frequency, but this was largely confined to complex methodological descriptions of a research design and procedure.

**Corson *et al.*** (Fertil Steril. 2005;83(4):855-6) defined and discussed the various forms taken by duplicate publications, and suggested remedies to help authors, editors, reviewers, and readers to avoid this form of internal plagiarism.

### Fabricated Data and Falsifying

**Stewart *et al.*** (Nature1987;325(6101):207-14) reported a case of admitted scientific fraud that has shed new light on the system that ensures the integrity of the scientific literature. Lapses from generally accepted standards of research may be more frequent than is commonly believed.

**Falagas *et al.*** (Arch Immunol Ther Exp (Warsz) 2008;56(4):223-6) observed that a considerable part of the scientific community is, at least to some degree, involved in the “impact factor game”. Editors strive to increase their journals' impact factor (IF) in order to gain influence in the fields of basic and applied research and scientists seek to profit from the “added value” of publishing in top IF journals. In this article they pointed out the most common “tricks” of engineering and manipulating the IF undertaken by a portion of professionals of the scientific publishing industry. They attempted to increase the nominator or decrease the denominator of the IF equation by taking advantage of certain design flaws and disadvantages of the IF that permit a degree of artificial and arbitrary inflation. Some of these practices, if not scientifically unethical, are at least questionable and should be abandoned. Editors and publishers should strive for quality through fair and thoughtful selection of papers forwarded for peer review and editorial comments that enhance the quality and scientific accuracy of a manuscript.

### Idea stealing and Previous ideas (Self plagiarism)

**Stark *et al.*** (Memory 2007;15(7):776-83) have opined that unconscious plagiarism (UP) occurs when an individual claims a previously experienced idea as their own. Previous studies have explored the cognitive precursors of such errors by manipulating the ways that ideas are thought about between initial idea exposure and later test. While imagining other's ideas does not increase rates of UP relative to control on either a recall-own or generate-new task, improving others' ideas substantially increases such errors in the recall-own task. This study explored the effects of elaboration on rates of UP when a source-monitoring test replaced the recall-own test. Plagiarism was again observed following idea improvement but not idea imagery even though participants engaged explicit source evaluation. Thus the probability of plagiarising another's idea appears linked to the generative nature of the idea processing performed.

**Bouville *et al.*** (Sci Eng Ethics 2008;14(3):311-22) are of the opinion that plagiarism is a crime against academics. It deceives readers, hurts plagiarized authors, and gets the plagiarist undeserved benefits. However, even though these arguments do show that copying other people's intellectual contribution is wrong, they do not apply to the copying of words. Copying a few sentences that contain no original idea (e.g. in the introduction) is of marginal importance compared to stealing the ideas of others. The two must be clearly distinguished, and the ‘plagiarism’ label should not be used for deeds which are very different in nature and importance.

### Copyright Infringement

**Hein** (J Biocommun. 1976;3(3):29-32) reported that institutional developers of mediated instruction for the health sciences in higher education must take whatever steps that are reasonable and necessary to obtain copyright protection for their original works and avoid liability for infringement. Twelve questions frequently asked by such developers in these two areas were discussed. Special requirements were set forth pertaining to material copyrightable by the developer, copyrighted by others, and in the public domain (not copyrightable by anyone). Unique requirements for writings, sound recordings and visual products were summarized. Relevant aspects of fair use, pre-publication copyright, post-publication copyright, and marketing and distribution through the private sector were set forth together with the elements of proof in infringement actions.

**Miller *et al.*** (Int J Instr Media. 1977-1978;5(1):1-8) reported four copyright infringement cases that significantly influenced the understanding of “fair use” copying, as it applies to educators and educational institutions.

### Ethics

**Drummond *et al.*** (J Physiol. 2009;587:713-9) reported the basic principles and methods that should be used regarding ethical matters in publication of manuscripts. They have summarized the UK law and the structure of regulations, and introduces the concept of research governance. They have given advice on the format and description of experiments and ethical considerations of publication such as authorship and originality, and problems such as plagiarism and fabrication.

**Reyes** (Rev Med Chil.2007;135(4):529-33) have opined that medical research must obey specific ethical rules that apply to studies involving human subjects, including biological samples, tissues, cellular or sub cellular samples obtained from them. When submitting their reports for publication, authors must declare that they have followed such ethical rules and also should declare any possible conflict of interest that may have arisen. External peer reviewers and the editors should also conform to limitations by eventual conflicts of interest. Authors should respect specific ethical norms that apply to the process of submitting, publishing and reproducing their manuscripts. In recent years, the editors of Revista Medica de Chile have become aware of five instances of misconduct committed by authors of articles submitted or already published. Four corresponded to redundant publications and one exhibited overt plagiarism in the text and syntax. Appropriate actions have been taken following recommendations published by the International Committee of Medical Journal Editors, the World Association of Medical Editors and other groups. The present article stressed that authors and their sponsoring institutions must be aware of the importance of following ethical rules when reporting scientific work.

**Coultas** (Proc Am Thorac Soc. 2007;4(2):194-8) is of the opinion that the ethical interpretation and communication of research results is essential to ensure the validity, timeliness, and accessibility of new knowledge for patients, physicians, and regulatory agencies. Failure to adhere to ethical principles may cause adverse outcomes for patients because of overestimation of benefit, underestimation of harm, and lack of timely awareness of benefit or harm. Although fabrication, falsification, and plagiarism are the traditional criteria for research misconduct, other more subtle behaviors may cause greater threats to public safety and trust in the research enterprise. Growing awareness of research misconduct has led to a number of initiatives worldwide during the past decade in an attempt to control the problem at various stages of the research process through the funding agencies, research institutions, and editorial oversight. The objective of this article was to raise awareness among the pulmonary research community of the broad range of ethical issues that arise during manuscript preparation, review, publication, and dissemination of research results, and efforts that are in progress to minimize misconduct.

**Benos *et al.*** (Adv Physiol Educ. 2005;29(2):59-74) summarized the major categories of ethical violations encountered during submission, review, and publication of scientific articles. They discussed data fabrication and falsification, plagiarism, redundant and duplicate publication, conflict of interest, authorship, animal and human welfare, and reviewer responsibility. In each section, pertinent historical background and citation of relevant regulations and statutes were provided.

### Misconduct: How to avoid ?

Research misconduct is defined by the Royal College of Physicians of Edinburgh as any behaviour by a researcher, whether intentional or not, that fails to scrupulously respect high scientific and ethical standards. Various types of research misconduct include fabrication or falsification of data, plagiarism, problematic data presentation or analysis, failure to obtain ethical approval by a research ethics committee or to obtain the subject's informed consent, inappropriate claims of authorship, duplicated publication, and undisclosed conflicts of interest. Pitak-Arnnop et al. (J Chir (Paris) 2008;145(6):534-41) studied these misconducts and reported that these can result in patient injury, deterioration of the patient-physician relationship, loss of public trust in biomedical research, and pollution/degradation of medical literature.

**Errami *et al.*** (Nucleic Acids Res.2009;37 (Database issue):D921-4) have made available Deja vu, a publicly available database of highly similar Medline citations identified by the text similarity search engine eTBLAST. Following manual verification, highly similar citation pairs have been classified into various categories ranging from duplicates with different authors to sanctioned duplicates. Deja vu records also contain user-provided commentary and supporting information to substantiate each document's categorization. Deja vu and eTBLAST are available to authors, editors, reviewers, ethicists and sociologists to study, intercept, annotate and deter questionable publication practices. These tools are part of a sustained effort to enhance the quality of Medline as the biomedical corpus.

**Bilic-Zulle *et al.*** (Sci Eng Ethics. 2008;14(1):139-47) performed a study to evaluate the effectiveness of plagiarism detection software and penalty for plagiarizing in detecting and deterring plagiarism among medical students. The study was a continuation of previously published research in which second-year medical students from 2001/2002 and 2002/2003 school years were required to write an essay based on one of the four scientific articles offered by the instructor. Students from 2004/2005 (N = 92) included in present study were given the same task. Topics of two of the four articles were considered less complex, and two were more complex. One less and one more complex articles were available only as hardcopies, whereas the other two were available in electronic format. The students from 2001/2002 (N = 111) were only told to write an original essay, whereas the students from 2002/2003 (N = 87) were additionally warned against plagiarism, explained what plagiarism was, and how to avoid it. The students from 2004/2005 were warned that their essays would be examined by plagiarism detection software and that those who had plagiarized would be penalized. Students from 2004/2005 plagiarized significantly less of their essays than students from the previous two groups (2% vs. 17% vs. 21%, respectively, P < 0.001). Over time, students more frequently selected articles with more complex subjects (P < 0.001) and articles in electronic format (P < 0.001) as a source for their essays, but it did not influence the rate of plagiarism. Use of plagiarism detection software in evaluation of essays and consequent penalties had effectively deterred students from plagiarizing.

**Wager *et al.*** (Med Law 2007;26(3):535-44) tried to discover what editors actually do when faced with cases of suspected scientific misconduct using cases submitted to the Committee on Publication Ethics (COPE). Of the 79 cases referred to COPE between 1998 and 2003 relating to author misconduct, 33 related to redundant publication, 16 to unethical research, 13 to fabrication, 10 to clinical misconduct and 7 to plagiarism. Outcomes were reported in 49 cases. Authors were exonerated in 16 cases and reprimanded in another 17. An impasse (no or an unsatisfactory response) was reached in 16. Editors contacted the authors' institutions in 24 cases. Nearly half the cases (36) lasted over a year. This small survey highlighted the difficulties faced by editors in pursuing cases of suspected misconduct and the need for better training and guidance for editors and more cooperation from institutions

**Wager *et al.*** (Menopause Int. 2007;13(3):98-102) described various types of publication misconduct and offerred guidance to authors, reviewers and journal editors about ways to detect and prevent them. Publication misconduct includes a range of unethical behaviours, such as plagiarism, breach of confidence and in appropriate authorship. The most egregious cases are easy to recognize and widely condemned, but the gradient between normal and unethical behaviour is often a gradual one. They appealed that clinicians and researchers should be aware of the full spectrum of publication misconduct and understand that some widely accepted practices may be unethical.

**Triggle *et al.*** (Vasc Health Risk Manag. 2007;3(1):39-53) reviewed the abuse of peer review and the method of policing it. A bad peer review process can inadvertently ruin an individual's career, but are there penalties for policing a reviewer who deliberately sabotages a manuscript or grant? Science has received an increasingly tainted name because of recent high profile cases of alleged scientific misconduct. Once considered the results of work stress or a temporary mental health problem, scientific misconduct is increasingly being reported and proved to be a repeat offence. How should scientific misconduct be handled--is it a criminal offence and subject to national or international law? Similarly plagiarism is an ever-increasing concern whether at the level of the student or a university president. Are the existing laws tough enough? These issues, with appropriate examples, were dealt with in this review.

**Pollard** (Best Pract Res Clin Anaesthesiol. 2006;20(4):653-68) emphasized the importance of ethics committee and its role in approval of a research project. The committees concern themselves with research but the differences between audit and research are difficult to discern in many places. If there is any doubt then the advice of the local research ethics committee should be sought. He opined that publication of results thought to be of lesser importance may prove difficult, however, and so there is a temptation to falsify or modify data to make it more attractive. This, together with other activities such as the fabrication of data, plagiarism, dual publication, salami publication, conflicts of interest and irregularities in authorship, have given editors of journals a number of problems.

**Scanlan.** (J Allied Health 2006;35(3):179-85) observed that student academic misconduct has become a growing problem for colleges and universities, including those responsible for preparing health professionals. Although the implementation of honour codes has had a positive impact on this problem, further reduction in student cheating and plagiarism can be achieved only via a comprehensive strategy that promotes an institutional culture of academic integrity. Such a strategy must combine efforts both to deter and detect academic misconduct, along with fair but rigorous application of sanctions against such behaviours. Methods useful in preventing or deterring dishonest behaviors among students include early integrity training complemented with course-level reinforcement, faculty role-modeling, and the application of selected testing/assignment preventive strategies, including honour pledges and honesty declarations. Giving students more responsibility for oversight of academic integrity also may help address this problem and better promote the culture needed to uphold its principles.

**Gollogly and Momen** (Rev Saude Publica.2006;40 Spec no.:24-9) discussed the definition of scientific misconduct, ways to document the extent of the problem, and examples of editorial attempts to counter fraud. Editorial misconduct includes failure to observe due process, undue delay in reaching decisions and communicating these to authors, inappropriate review procedures, and confounding a journal's content with its advertising or promotional potential. He added that editors can be admonished by their peers for failure to investigate suspected misconduct, failure to retract when indicated, and failure to abide voluntarily by the six main sources of relevant international guidelines on research, its reporting and editorial practice. Editors are in a good position to promulgate reasonable standards of practice, and can start by using consensus guidelines on publication ethics to state explicitly how their journals function. Reviewers, editors, and authors all then have a better chance to understand, and abide by, the rules of publishing.

**Kvaal** (J Am Coll Dent. 2008;75(2):29-35) reported that in 2006 a researcher at the main hospital in Norway admitted that he had forged data in a study published in the medical journal ‘The Lancet’ that was co-authored by 13 others from both Europe and America. The researcher, dually qualified in dentistry and medicine, immediately admitted fabricating the results. A Commission of Enquiry reported that most of his publications were fabricated or manipulated and that he was alone in the fraud. As a result, the researcher lost his authorization to practice medicine and dentistry. Following this revelation, the management of scientific fraud has been widely discussed, including concerns about the dual role of a Commission of Enquiry as both investigator and judge, and also the legal rights of fraudulent scientists. Other issues concern the responsibilities of supervisors and institutions in the guidance of candidates in research procedures and ethics. Various issues have been discussed, including the fact that editors and referees in scientific publications rarely have the opportunity to check raw data, which emphasizes the need for data confirmation by independent groups.

**Qamra *et al.*** (IEEE Trans Pattern Anal Mach Intell. 2005;27(3):379-91) observed that the proliferation of digital images and the widespread distribution of digital data that has been made possible by the internet has increased problems associated with copyright infringement on digital images. Watermarking schemes have been proposed to safeguard copyrighted images, but watermarks are vulnerable to image processing and geometric distortions and may not be very effective. Thus, the content-based detection of pirated images has become an important application. In this paper, they discussed two important aspects of such a replica detection system: distance functions for similarity measurement and scalability. They extended their previous work on perceptual distance functions, which proposed the Dynamic Partial Function (DPF), and present enhanced techniques that overcome the limitations of DPF. These techniques included the Thresholding, Sampling, and Weighting schemes. Experimental evaluations showed superior performance compared to DPF and other distance functions. They then addressed the issue of using these perceptual distance functions to efficiently detect replicas in large image data sets. The problem of indexing is made challenging by the high-dimensionality and the nonmetric nature of the distance functions. They proposed using Locality Sensitive Hashing (LSH) to index images while using the above perceptual distance functions and demonstrated good performance through empirical studies on a very large database of diverse images.

### Misconduct or Mistake

**Nath *et al.*** (Med J Aust.2006;185(3):152-4) performed a study to determine how commonly articles are retracted on the basis of unintentional mistakes, and whether these articles differ from those retracted for scientific misconduct in authorship, funding, type of study, publication, and time to retraction. Of the 395 articles retracted between 1982 and 2002, 107 (27.1%) were retracted because of scientific misconduct, 244 (61.8%) because of unintentional errors, and 44 (11.1%) could not be categorised. Compared with articles retracted because of misconduct, articles with unintentional mistakes were more likely to have multiple authors, no reported funding source, and to be published in frequently cited journals. They were more likely to be retracted by the author(s) of the article, and the retraction was more likely to occur more promptly (mean, 2.0 years; 95% CI, 1.8-2.2) than articles withdrawn because of misconduct (mean, 3.3 years; 95% CI, 2.7-3.9) (P < 0.05 for all comparisons).

**Neill** (J Clin Invest.2008;118(7):2368) observed that the academic scientific enterprise rewards those with the longest CVs and the most publications. Under pressure to generate voluminous output, scientists often fall prey to double publishing, self plagiarism, and submitting the “minimal publishable unit.”

